# Path-Dependent Constitutive Modeling for Superplastic Forming of Titanium Alloys: Memory-Architecture Perspective

**DOI:** 10.3390/ma19132817

**Published:** 2026-07-02

**Authors:** Ling Ding, Cik Suhana Hassan, Wei Hong Lim, Swee Pin Yeap, Ke Wei, Lu-Cui Chao

**Affiliations:** 1 Faculty of Engineering, Technology and Built Environment, UCSI University, Kuala Lumpur 56000, Malaysia; 1002476803@ucsiuniversity.edu.my (L.D.); limwh@ucsiuniversity.edu.my (W.H.L.); yeapsw@ucsiuniversity.edu.my (S.P.Y.); 2 Faculty of Information and Intelligent Engineering, Yunnan College of Business Management, No. 17 Qilin Road, Vocational Education Park, Anning 650300, China; 20122025@ynjgy.edu.cn; 3 Faculty of Materials Science and Engineering, Nanchang Hangkong University, Nanchang 330063, China; weike@nchu.edu.cn

**Keywords:** superplastic forming, titanium alloys, path-dependent constitutive modeling, memory architecture, internal state variables

## Abstract

Superplastic forming (SPF) of titanium alloys exhibits strong deformation path dependence because microstructural evolution, damage development, and material response are influenced by prior loading history. However, constitutive models for SPF are often evaluated primarily by fitting accuracy rather than their ability to represent deformation history. This review examines path-dependent constitutive modeling from a memory-architecture perspective. The relevant literature was identified through a structured review of titanium-alloy SPF studies, which were supplemented by selected high-temperature forming studies from other metallic systems when they provided transferable constitutive frameworks. Existing constitutive models were classified into four categories according to how deformation history is retained and represented: stateless models, implicit or projected memory models, reduced-order memory models, and high-dimensional or explicit memory models. The analysis shows that many conventional formulations achieve acceptable accuracy within calibrated monotonic regimes by strongly compressing deformation history, thereby limiting their ability to distinguish complex loading paths. Internal-state-variable models provide a practical balance between path representation, interpretability, and implementation, whereas high-dimensional memory models offer stronger sequence sensitivity at the cost of greater data and calibration requirements. This memory-architecture framework clarifies the limitations and applicability of existing constitutive models and provides guidance for model selection in SPF process simulation.

## 1. Introduction

### 1.1. Titanium-Alloy SPF and the Importance of Deformation History

Superplastic forming (SPF) of titanium alloys is an important manufacturing route for producing complex, near-net-shape components for aerospace and other high-performance engineering applications. Titanium alloys combine high specific strength, corrosion resistance, and thermal stability, while superplastic deformation enables exceptionally large elongations at elevated temperatures and low strain rates, allowing complex geometries to be manufactured with reduced machining and material waste [1,2,3].

The mechanical response of titanium alloys under SPF conditions is strongly influenced by deformation history. During high-temperature deformation, grain boundary sliding, dislocation accommodation, dynamic recovery, dynamic recrystallization, phase transformation, grain growth, and cavitation evolve continuously and interact with one another throughout the deformation process [3,4,5,6,7]. As a result, the material response at a given instant is not determined solely by the current strain, strain rate, or temperature but also by the preceding thermo-mechanical history.

The consequences of this history dependence are evident at the process level. Experimental studies involving variable strain-rate deformation, maximum-*m* forming, and multi-stage deformation strategies have shown that distinct loading schedules can produce substantially different flow behavior and formability, even under otherwise comparable processing conditions [8,9,10]. These observations suggest that superplastic deformation should be viewed as a history-sensitive process, in which the predictive capability of constitutive models depends not only on their ability to describe instantaneous material behavior but also on how effectively prior thermo-mechanical information is represented within the constitutive formulation.

### 1.2. Research Gap and Rationale for a Memory-Architecture Perspective

Constitutive models provide the foundation for process simulation, forming-path design, thickness prediction, and optimization of SPF operations. Traditional constitutive formulations, including power-law, hyperbolic-sine, Arrhenius-type, and Johnson–Cook-type models, remain widely used because of their simplicity, computational efficiency, and relatively low calibration cost [2,11,12]. To improve the representation of evolving material behavior, physically based constitutive formulations have incorporated microstructure-related variables such as grain size, dislocation density, recrystallization fraction, phase fraction, and damage descriptors [7,13,14]. More recently, data-driven and hybrid approaches have further expanded the range of constitutive modeling strategies available for high-temperature deformation and SPF applications [15,16].

Despite these advances, existing reviews and model comparisons generally classify constitutive models according to their mathematical formulation, empirical or physical basis, alloy system, or prediction accuracy. Such classifications are valuable for describing model characteristics and application domains, but they provide limited insight into a fundamental issue underlying path-dependent deformation: how information from prior deformation history is represented within the constitutive formulation itself.

This limitation becomes particularly important in SPF, where material response is influenced by evolving microstructure, damage development, and thermo-mechanical loading history [7,13,17,18]. Under variable strain-rate conditions, multi-stage forming schedules, or other history-sensitive deformation paths, constitutive models with similar fitting accuracy may exhibit substantially different predictive capabilities [6,8,9,10]. However, the relationship between deformation-history representation and constitutive performance remains insufficiently discussed in the existing literature.

The central gap addressed in this review is therefore the lack of a systematic framework for examining constitutive models from the perspective of deformation-history representation. Existing classifications provide limited guidance regarding the extent to which prior loading information is retained, transformed, or utilized by different constitutive formulations, and how these differences influence predictive capability under complex deformation paths.

To address this gap, the present review introduces a memory-architecture perspective for analyzing constitutive models used in titanium-alloy SPF and related high-temperature deformation processes. Rather than focusing primarily on model origin or mathematical form, this perspective examines how deformation history is represented within constitutive formulations and how such representations influence path-dependent prediction. By adopting this viewpoint, a common framework can be established for comparing empirical, physically based, and data-driven constitutive approaches.

### 1.3. Scope and Review Methodology

This review focuses primarily on titanium alloys under superplastic forming (SPF) and related high-temperature deformation conditions. The evidence base therefore consists mainly of studies addressing constitutive modeling, flow behavior, and microstructure-sensitive deformation responses in titanium alloys.

Because the objective of this review is conceptual framework development rather than exhaustive evidence synthesis, the literature selection process was designed to identify representative constitutive architectures rather than to establish a complete census of all SPF constitutive studies. Studies were therefore selected according to their relevance to deformation-history representation and constitutive memory structures, while highly repetitive variants of similar constitutive formulations were consolidated to avoid redundancy.

The literature survey was conducted primarily using the Web of Science Core Collection database through a three-stage screening strategy. First, studies related to titanium-alloy SPF and high-temperature constitutive modeling were collected, yielding more than 400 records after preliminary filtering. Second, concepts commonly associated with deformation-history representation, including path dependence, internal-state evolution, microstructural evolution, and memory-related descriptors, were used to identify potentially relevant studies, reducing the dataset to approximately 200 records for detailed assessment. Full-text evaluation showed that only a limited subset explicitly incorporated deformation-history retention into the constitutive architecture.

Because the titanium-alloy literature alone provided only partial coverage of the constitutive memory architectures considered in this review, the material restriction was selectively relaxed in a third-stage search. This search yielded more than 300 additional records from other metallic systems, from which a small number of representative studies were retained to provide transferable examples of internal-state-variable, physically based, and sequence-aware constitutive frameworks.

After full-text assessment, removal of redundant model variants, and targeted supplementation through citation tracking, approximately 85 representative publications were retained as the evidence base for the present review. Earlier landmark studies were additionally included when necessary to establish the historical development of constitutive modeling concepts and memory-representation strategies.

The objectives of this review are therefore to: (i) establish a memory-architecture framework for constitutive models used in titanium-alloy SPF; (ii) classify existing constitutive formulations according to how deformation history is represented and retained; (iii) evaluate the relationship between memory capacity, path distinguishability, and predictive capability; (iv) discuss the implications of constitutive memory architecture for constitutive-model selection and SPF process simulation.

## 2. Conceptual Foundations of Path-Dependent Constitutive Modeling for SPF

This section establishes why deformation history must be considered in constitutive modeling for titanium-alloy SPF. The purpose is not to provide an exhaustive mechanistic review of superplasticity but to identify the microstructural features that make the macroscopic response non-unique with respect to instantaneous strain, strain rate, and temperature. These features define the physical requirements that motivate the memory-architecture classification developed in Section 3.

### 2.1. Microstructural Origins of Path Dependence in Superplastic Deformation

Superplastic deformation in titanium alloys is governed by irreversible and history-sensitive microstructural evolution. Grain boundary sliding is widely recognized as a dominant deformation mode in fine-grained titanium alloys under suitable SPF conditions, but its continuity depends on accommodation by intragranular dislocation activity, diffusion-assisted processes, boundary migration, and microstructural rearrangement [19,20,21,22,23]. As deformation proceeds, dislocations accumulate and recover, grain boundaries migrate, and local accommodation structures are progressively reconstructed. The instantaneous flow response therefore reflects not only the current loading condition but also the internal state created by earlier deformation increments.

Dynamic recovery and dynamic recrystallization introduce particularly strong sources of irreversibility. Recrystallization nucleation is influenced by stored energy accumulated during prior dislocation activity, while grain growth, grain refinement, and changes in boundary topology modify later hardening and softening behavior [13,17,24,25]. In two-phase titanium alloys, α/β phase transformation, strain partitioning, and evolving phase morphology further amplify path dependence by changing load-sharing and accommodation pathways [3,13,26]. These mechanisms imply that the same nominal strain can correspond to different microstructural states if it is reached through different temperature or strain-rate histories.

**Figure 1:**
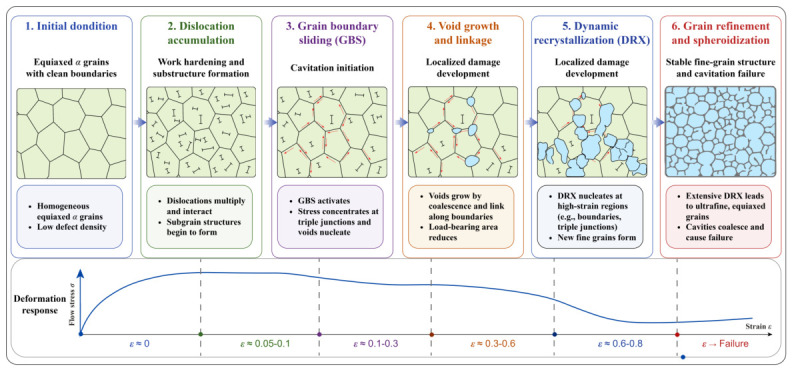
Schematic representation of microstructural evolution during superplastic deformation of Ti60 alloy, including phase transition, dislocation rearrangement, grain boundary sliding, recovery, and recrystallization. The red arrows along grain boundaries indicate the direction of grain boundary sliding. The black schematic symbols represent dislocations within grains. Redrawn from Li et al. [1].

The staged nature of these mechanisms is illustrated schematically in Figure 1 [1]. The figure summarizes a progressive transition in mechanism dominance, from early dislocation accumulation and phase-related changes to grain boundary sliding, recovery, and recrystallization-mediated softening. The key point for constitutive modeling is that the stress at a given strain reflects a microstructure that has been cumulatively constructed through the preceding thermo-mechanical path.

**Figure 2:**
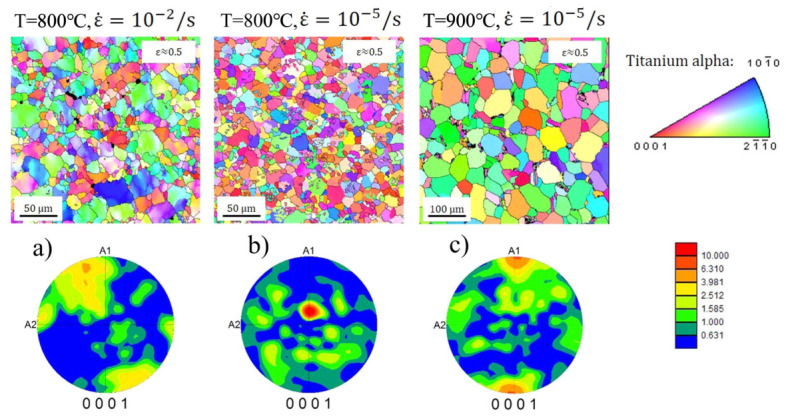
Representative EBSD maps and corresponding pole figures of TA15 alloy deformed to comparable strain levels (ε≈0.5) under different thermo-mechanical conditions. Subfigures (**a**–**c**) correspond to deformation at 800 °C/10^−2^ s^−1^, 800 °C/10^−5^ s^−1^, and 900 °C/10^−5^ s^−1^, respectively. Despite similar accumulated strain, substantial differences in grain morphology and grain-size distribution are observed, demonstrating the non-uniqueness of strain-equivalent states and highlighting the history-sensitive nature of superplastic deformation. Adapted from Alabort et al. [17].

Direct microstructural evidence of path-sensitive state construction is provided by EBSD observations under different thermo-mechanical conditions. As shown in Figure 2 [17], TA15 specimens deformed under different temperatures and strain rates can exhibit distinct grain morphologies, recrystallization fractions, and grain-size distributions even when compared at similar accumulated strains. These observations demonstrate that strain-equivalent states need not be microstructurally equivalent. For modeling purposes, this means that accumulated strain alone is an incomplete descriptor of deformation history.

### 2.2. Structural Necessity of Path Encoding in Constitutive Modeling

The microstructural mechanisms discussed in the previous section establish that superplastic deformation is inherently history sensitive. For constitutive modeling, however, the central challenge is not merely the existence of path dependence, but how information associated with prior deformation is represented within the constitutive formulation. Experimental and modeling studies have shown that different thermo-mechanical paths may lead to distinct material responses even when the current deformation conditions appear similar [17,27,28]. Consequently, constitutive modeling must address the problem of representing deformation history in a form that can influence the current material state and response.

In principle, the constitutive response at a given instant can be viewed as a functional of the entire preceding thermo-mechanical history. Let $H_{t}$ denote the deformation history accumulated up to time *t*. The constitutive response may then be expressed conceptually as:

(1) $$\sigma \left(t\right)=F\left(H_{t}\right)$$

where $H_{t}$ represents the complete deformation history and F denotes the constitutive mapping from history to material response. Such a formulation is conceptually general, but it is impractical for engineering applications because the amount of information contained in the full deformation history grows continuously throughout the forming process.

A constitutive model therefore faces an unavoidable information-reduction problem. Instead of retaining the complete deformation history, the model must construct a reduced representation that preserves only the information considered relevant to future material behavior. This process may be represented conceptually as:

(2) $$H_{t}\to M_{t}\to \sigma \left(t\right)$$

where $M_{t}$ denotes the memory representation available to the constitutive model at time *t*. The specific form of $M_{t}$ may vary considerably depending on the constitutive formulation. Historical information may be discarded entirely, compressed into accumulated descriptors, retained through evolving internal states, or represented through more complex physical or computational structures [7,13,17,29,30].

From this perspective, constitutive modeling may be viewed as a process of history representation rather than merely stress prediction. The predictive capability of a constitutive model under complex loading conditions depends not only on its mathematical form but also on the amount and type of historical information that remains available to the model during deformation. Two constitutive formulations may therefore achieve similar fitting accuracy under monotonic loading while possessing substantially different abilities to distinguish alternative deformation paths [27,30,31].

This observation suggests that path dependence should be examined at the level of constitutive information architecture. The central question is not whether a model is empirical, physically based, or data driven, but rather how deformation history is represented within the constitutive structure itself. This viewpoint provides the conceptual foundation for the memory-architecture framework developed in the following section.

## 3. Path-Dependent Constitutive Modeling Frameworks

### 3.1. Memory-Architecture-Based Classification

Based on the memory-architecture perspective introduced in Section 2, four broad classes of constitutive models are distinguished according to how deformation history is represented within the constitutive structure.

For clarity, the generic constitutive forms listed in Table 1 use three abstract memory representations:

*P* denotes projected descriptors through which deformation history is compressed into fitted quantities, typically strain-dependent parameters; *S* denotes a finite set of independently evolving internal state variables; *M* denotes high-dimensional memory representations, including latent sequence states, physical fields, or other rich state structures.

The defining characteristics of each class are summarized in Table 1.

The following subsections examine each of these four architectures in order of increasing memory capacity, from stateless formulations to high-dimensional memory models.

### 3.2. Implicit or Projected Memory Models

Implicit or projected memory models denote constitutive formulations in which deformation history is not retained through independently evolving state variables. Instead, historical information is compressed into instantaneous constitutive descriptors, most commonly strain-dependent parameters or coupled thermo-mechanical quantities. The defining feature of this architecture is that deformation history influences the constitutive response only through its effect on current parameter values. Consequently, the model retains a limited representation of prior deformation while avoiding explicit state evolution.

#### 3.2.1. Forms of History Projection

The most common form of projected memory is strain-based parameter evolution. In strain-compensated Arrhenius formulations, constitutive parameters such as the stress exponent, activation energy, stress multiplier, and prefactor are expressed as functions of accumulated strain, typically through polynomial fitting or interval-wise calibration procedures [27,39,40,41,42,43,44,45,46,47,48,49]. Similar approaches are widely adopted in modified Arrhenius and strain-ratio-based formulations, where strain-dependent scaling functions are introduced to improve the description of strain hardening and softening behavior without introducing independent state variables [22,50,51].

Projection can also be distributed across multiple constitutive parameters. In generalized Arrhenius formulations, modified Johnson–Cook models, and related phenomenological constitutive equations, quantities such as activation energy, stress exponent, hardening exponent, and thermal softening parameters evolve simultaneously with strain and thermo-mechanical conditions [34,47,52,53]. Although the constitutive representation becomes more flexible, the historical information remains embedded within parameter values rather than retained through independently evolving state variables.

A further extension incorporates process-specific or stress-state descriptors. Examples include constitutive formulations involving stress triaxiality, Lode angle, electric current density, machining parameters, or other coupled process variables [54,55,56,57,58,59]. Similar concepts appear in forming-process studies where loading conditions influence constitutive calibration or process response [60]. Despite differences in implementation, all of these approaches share the same architectural principle: deformation history is represented indirectly through instantaneous descriptors rather than through an explicit memory state.

#### 3.2.2. Predictive Capability and Structural Limitations of Projected-Memory Models

Projected-memory models have demonstrated excellent predictive performance under monotonic deformation paths and within calibrated processing windows. Strain-compensated Arrhenius formulations, modified Arrhenius equations, and Johnson–Cook-type models consistently provide improved fitting accuracy compared with fully stateless formulations, particularly for describing strain hardening, flow softening, and steady-state flow behavior under proportional loading conditions [27,31,34,44]. In most studies, validation is performed using constant strain-rate and temperature tests, and model quality is evaluated primarily through flow-stress prediction accuracy [42,43,47,49].

From a memory-architecture perspective, however, these predictive gains arise from history projection rather than history retention. Because prior deformation is compressed into instantaneous descriptors, different thermo-mechanical paths that converge to the same descriptor values become constitutively indistinguishable. This limitation is structural rather than a consequence of calibration quality. As a result, projected-memory models are well suited for interpolation within calibrated monotonic regimes, but their ability to represent strain-rate jumps, thermal transients, multi-stage loading, or other sequence-sensitive deformation paths remains inherently constrained. These limitations motivate constitutive frameworks that retain deformation history through independently evolving memory states.

### 3.3. Reduced-Order Memory Models

Reduced-order memory models retain deformation history through a finite set of explicitly evolving internal state variables that are directly coupled to the constitutive response. Rather than storing the full deformation sequence, these models represent history through selected state descriptors intended to capture the mechanically relevant consequences of prior loading. Typical examples include dislocation density, grain size, recrystallized fraction, phase fraction, and damage-related variables, each governed by prescribed evolution laws [7,18,36,61,62]. Compared with projected-memory models, deformation history is not embedded in fitted parameters but retained in the current values of independently evolving state variables.

#### 3.3.1. Internal-State Representation of Deformation History

The central assumption underlying reduced-order memory models is that the effects of deformation history can be represented by a low-dimensional internal state vector. Through incremental evolution laws, variables such as grain size, dislocation density, recrystallized fraction, phase fraction, and damage indicators continuously accumulate information associated with strain, strain rate, temperature, and other loading factors [6,35,63]. The constitutive response therefore depends not only on the instantaneous loading condition but also on the current internal state generated by prior deformation.

This strategy is widely adopted in unified viscoplastic formulations, grain-size evolution models, dynamic recrystallization models, damage and cavitation models, and multi-phase constitutive frameworks [7,18,36,62,64,65]. Although the physical interpretation of individual state variables differs among formulations, they share the same architectural principle: deformation history is retained in a compressed but explicitly evolving state space. Internal variables enter the constitutive response directly through hardening, softening, phase-transformation, grain-growth, or damage-related mechanisms, enabling the model to represent microstructure-sensitive and partially sequence-dependent behavior [36,62,66].

Because state evolution is integrated incrementally through time, reduced-order memory models are inherently capable of representing path dependence. Studies involving strain-rate jumps, loading-path changes, cumulative shear deformation, and transformation-induced effects have shown that different loading histories can produce different internal states and stress responses even at identical strain levels [6,7,35,67,68]. However, in many formulations, path dependence is architecturally present but only partially exercised, as validation is commonly performed under monotonic deformation at different strain rates or temperatures rather than under explicitly path-distinguishing loading schedules [4,61,69,70].

#### 3.3.2. Predictive Capability and Architectural Limitations of Reduced-Order Memory Models

Reduced-order memory models provide a practical balance between constitutive fidelity and model complexity. By explicitly coupling internal-state evolution with the constitutive response, these models can represent strain-rate sensitivity, thermal-history effects, microstructure-driven hardening and softening, dynamic recrystallization, phase evolution, damage accumulation, and selected classes of sequence-dependent behavior [35,62,67]. As a result, they are particularly attractive for SPF simulations in which microstructural evolution, cavitation, or formability prediction plays an important role.

From a memory-architecture perspective, however, the memory capacity of these models remains finite. As discussed in Section 2.2, the information-reduction problem implies that distinct loading paths may evolve toward similar internal states despite differences in their actual deformation histories. The predictive capability of reduced-order memory models consequently depends strongly on the selection of state variables and the adequacy of their evolution equations. While they represent a substantial advance beyond projected-memory approaches, their ability to distinguish highly complex, long-range, or strongly sequence-dependent deformation histories remains constrained by the reduced dimensionality of the internal state space.

### 3.4. High-Dimensional or Explicit Memory Models

High-dimensional or explicit memory models represent deformation history through rich state representations, including high-dimensional physical states, latent sequence states, and spatially resolved fields, which preserve substantially more information than the compressed representations of Type II and III architectures. The defining architectural characteristic of this class is expanded memory capacity, enabling the constitutive response to remain sensitive to loading order, temporal interactions, and complex deformation histories that may be indistinguishable under reduced-order representations.

#### 3.4.1. Forms of High-Dimensional Memory Representation

One route to high-dimensional memory is through physically explicit state spaces. Multi-variable dislocation-based models, morphology-resolved formulations, and micromechanics-based approaches retain history by simultaneously tracking large numbers of interacting microstructural variables, including multiple dislocation populations, grain and subgrain structures, boundary characteristics, phase fractions, and orientation-dependent quantities [37,71,72]. Although individual variables may be physically interpretable, the combined state space becomes high-dimensional and evolves recursively through coupled evolution laws. Deformation history is therefore retained in the collective state of the system rather than in a small set of reduced descriptors.

A second route is sequence-based memory learned directly from data. Recurrent neural networks, particularly LSTM-based constitutive models, encode deformation history through high-dimensional hidden states that are updated continuously as the loading sequence evolves [30,73,74,75,76]. More recent attention-based and Transformer-inspired architectures extend this concept by allowing the constitutive response to selectively attend to different portions of the deformation history [38,77]. In contrast to predefined internal-state variables, these latent representations are learned automatically from data and can preserve ordering, timing, and long-range interactions among prior deformation events.

Despite differences in implementation, both physically explicit and data-driven approaches share the same architectural principle: deformation history is retained in a rich memory representation whose dimensionality exceeds that of conventional internal-state-variable models. As a result, loading paths that collapse to similar descriptors or state vectors in lower-dimensional models may remain distinguishable within the high-dimensional memory space.

#### 3.4.2. Predictive Capability and Architectural Limitations of High-Dimensional Memory Models

High-dimensional memory models offer the strongest capacity for representing path dependence among the constitutive architectures considered in this review. Studies have demonstrated their ability to reproduce strain-rate jump effects, non-isothermal deformation, complex multi-stage loading histories, orthogonal hardening and softening, and other sequence-sensitive responses that are difficult to represent using projected or reduced-order memory structures [30,37,73,74]. Their flexibility also enables the integration of microstructural information, physical constraints, and data-driven learning within a unified constitutive framework [38,71].

From a memory-architecture perspective, however, increased memory capacity is accompanied by increased complexity. Physically explicit models require large numbers of state variables, extensive calibration data, and significant computational resources [71,72]. Data-driven architectures depend on path-resolved training datasets and often lack direct physical interpretability, making extrapolation beyond the training domain difficult to assess [75,76]. Moreover, despite their theoretical advantages, most published validations remain concentrated on monotonic or weakly varying loading paths, while applications involving strongly non-proportional SPF histories remain comparatively limited [38,77].

Consequently, high-dimensional memory models should be viewed not as universal replacements for reduced-order formulations, but as upper-bound representations of constitutive memory. Their principal value lies in demonstrating the extent to which path dependence can be retained when memory constraints are relaxed, thereby providing useful benchmarks and inspiration for the development of hybrid architectures that balance predictive capability, interpretability, and practical implementation in SPF simulations.

### 3.5. Architecture-Level Synthesis

**Figure 3:**
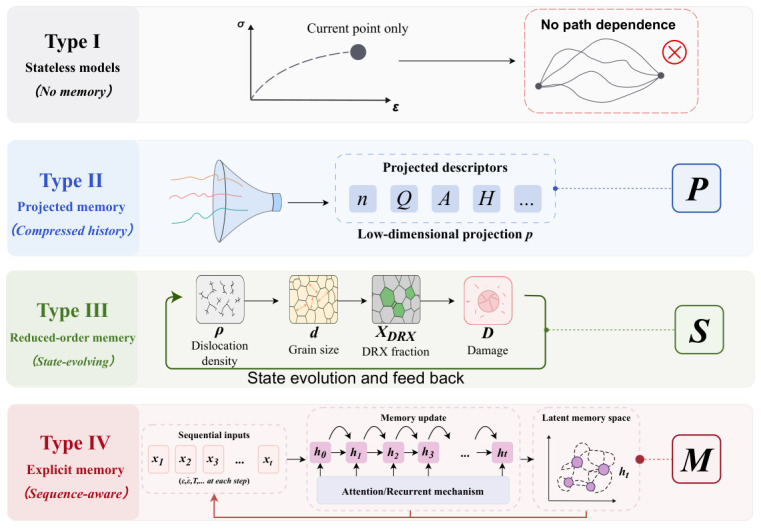
Conceptual representation of deformation-history retention across constitutive memory architectures. The schematic illustrates the progressive transition from stateless constitutive formulations to high-dimensional memory architectures, highlighting the increasing amount of deformation-history information retained and made available to influence constitutive response. Colors are used only to distinguish different constitutive memory architectures. Ellipses (“…”) indicate representative rather than exhaustive memory descriptors or sequential states.

Figure 3 summarizes the principal mechanisms through which deformation history is represented within the four constitutive memory architectures introduced in this review. The figure illustrates a progression from complete history elimination, through history projection and reduced-order state evolution, to high-dimensional memory representations capable of retaining richer information about prior deformation. Viewed from this perspective, constitutive models differ not only in their mathematical formulation but also in the amount of historical information retained and made available to influence future material response.

The practical implications of these architectural differences are summarized in Table 2. As memory capacity increases, constitutive models generally gain greater ability to represent path-dependent behavior, but this is accompanied by increasing calibration effort, data requirements, and implementation complexity. Consequently, no single architecture is universally optimal. The appropriate choice depends on the balance between predictive requirements and the cost associated with retaining additional deformation-history information.

While the specific implementations differ substantially, the four classes can be viewed as occupying different positions along a continuum of constitutive memory representation, providing a unified basis for comparing empirical, physically based, and data-driven constitutive approaches.

## 4. Discussion: Implications of Memory Architecture for SPF

The classification presented in Section 3 highlights that constitutive models differ primarily in how deformation history is represented. The practical importance of these differences depends on the prediction task. While some SPF applications require only an accurate description of the instantaneous flow response, others depend critically on the ability to track evolving material states and cumulative deformation effects. The following discussion therefore examines the relationship between constitutive memory and SPF process requirements, evaluates current modeling practices, and considers future directions for constitutive development.

### 4.1. Memory Capacity Versus SPF Process Requirements

For monotonic flow-stress prediction, Type I and Type II architectures are generally sufficient because the material follows a relatively simple thermo-mechanical trajectory and the constitutive response is governed primarily by the current strain rate and temperature. Classical power-law, Arrhenius-type, strain-compensated Arrhenius, and modified Johnson–Cook formulations have repeatedly demonstrated satisfactory predictive performance within calibrated processing windows [27,31,42]. Under such conditions, the deformation path contains limited additional information beyond the current loading state, and increasing constitutive memory capacity therefore provides relatively little practical benefit.

The requirements become more demanding when constitutive models are used for SPF process simulation. Thickness evolution, strain localization, and pressure-cycle optimization involve continuously changing deformation conditions in which local strain rate, stress state, and deformation history evolve throughout the forming process [7,17,62]. In these applications, the material response depends increasingly on the evolving internal state generated by prior deformation rather than on instantaneous loading conditions alone. Wang et al. demonstrated that an ISV-based constitutive model improved thickness prediction during hot gas pressure forming of welded titanium-alloy blanks by accounting for the evolution of dislocation density and damage during deformation [7]. Similarly, the strain-rate sensitivity index *m* evolves as microstructure changes during SPF, implying that optimal pressure schedules depend on the current material state rather than on nominal forming parameters alone [68]. These requirements favor constitutive architectures that explicitly retain deformation history through evolving state variables.

The strongest demand for constitutive memory arises when the prediction target itself is an evolving internal state. Dynamic recrystallization, grain growth, phase transformation, cavitation, and damage accumulation are cumulative processes whose evolution depends on the integrated effects of prior thermo-mechanical loading [6,13,17]. Different deformation histories may therefore produce distinct microstructural states even when they terminate at similar strain levels [17,78]. Constitutive formulations that represent history only through projected descriptors are structurally unable to describe such phenomena directly. Reduced-order memory architectures, particularly ISV-based formulations, provide a natural framework because deformation history is retained through variables such as grain size, recrystallized fraction, dislocation density, phase fraction, and damage indicators [6,13,18]. The ability to distinguish internal states generated by different loading histories consequently becomes essential for reliable prediction of microstructure evolution and damage development.

**Figure 4:**
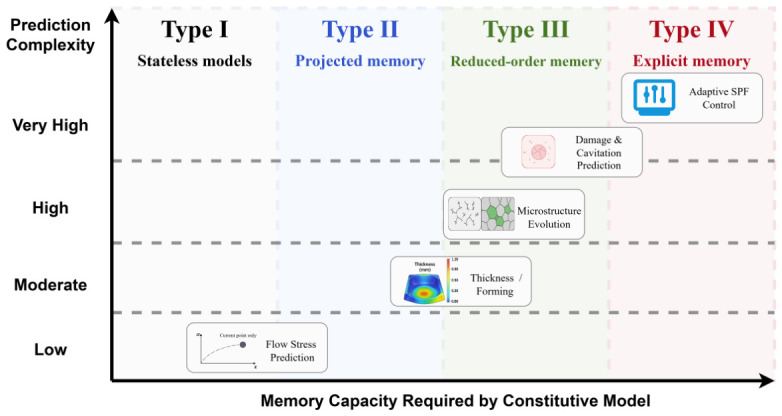
Memory-aware framework for selecting constitutive architectures according to SPF prediction requirements. The framework links representative SPF prediction tasks with constitutive memory architectures of increasing memory capacity, illustrating how the need for deformation-history retention grows from monotonic flow-stress prediction to microstructural evolution, damage prediction, and adaptive process-control applications.

Figure 4 provides a schematic framework linking representative SPF prediction tasks to the constitutive memory architectures that are generally most advantageous for their description. At the lowest tier, monotonic flow-stress prediction under constant strain-rate and temperature conditions can often be adequately represented using Type I–II architectures, where the deformation path contributes relatively little information beyond the instantaneous loading state. Thickness evolution and forming-path prediction under continuously changing deformation conditions increasingly benefit from Type II–III architectures, as the material response becomes more dependent on the evolving internal state generated by prior deformation. When the prediction target includes microstructural evolution, such as dynamic recrystallization, grain growth, or phase transformation, Type III architectures provide a natural framework through explicit internal-state variables, including grain size, recrystallized fraction, and dislocation density. Damage and cavitation prediction, which depends on the cumulative effects of prior thermo-mechanical loading, may benefit from Type III–IV architectures when greater representation of loading history and sequence effects is required. At the highest tier, adaptive SPF control applications involving real-time state estimation, process optimization, or strongly non-proportional loading histories can particularly benefit from the enhanced deformation-history retention capacity of Type IV architectures. This progressive mapping contextualizes the practical roles of the four memory architectures and supports memory-aware constitutive-model selection in SPF process simulation.

### 4.2. Critical Assessment of Current SPF Constitutive Modeling

Two broad observations emerge from the reviewed literature.

#### 4.2.1. Dominance of Accuracy-Oriented Modeling Strategies

Despite increasing recognition that superplastic deformation is fundamentally history dependent, most constitutive studies continue to rely on projected-memory formulations such as strain-compensated Arrhenius, modified Arrhenius, and Johnson–Cook-type models [27,34,47,53]. These approaches remain attractive because they are computationally efficient, relatively straightforward to calibrate, and capable of providing satisfactory predictive accuracy within calibrated processing windows.

Their continued dominance has influenced not only constitutive-model development but also the criteria used to evaluate constitutive performance. In much of the SPF literature, model quality is assessed primarily through agreement with monotonic flow-stress data, and improvements are commonly reported in terms of fitting accuracy, correlation coefficients, or prediction errors [31,42,44]. Such metrics are undoubtedly important for practical implementation, particularly in finite-element simulations and process optimization studies.

However, for path-dependent deformation processes, predictive accuracy and constitutive capability are not necessarily equivalent. A model may reproduce monotonic flow curves with high accuracy while providing limited information about how prior thermo-mechanical history influences subsequent material response. Consequently, constitutive fidelity is often implicitly equated with fitting performance, whereas the ability to represent deformation history is evaluated far less frequently. The widespread adoption of projected-memory formulations therefore reflects not only their practical advantages but also a broader tendency within the literature to prioritize predictive accuracy over explicit assessment of path-dependent behavior.

#### 4.2.2. Gap Between Memory Capacity and Demonstrated Path-Dependent Capability

A distinction should first be made between studies conducted directly on titanium-alloy SPF and studies drawn from other high-temperature deformation systems. Because explicitly path-distinguishing validation remains relatively uncommon in the titanium-SPF literature, evidence in this review is supplemented by selected investigations on aluminum and magnesium alloys, where similar history-sensitive deformation behavior, microstructural evolution, and constitutive-memory challenges have been examined. These studies are not used to infer titanium-alloy behavior directly, but rather to evaluate general principles of deformation-history representation and constitutive validation.

Many Type III and Type IV constitutive architectures are structurally capable of retaining deformation history through evolving internal states, latent sequence representations, or physically explicit state variables. In practice, however, direct evidence demonstrating path-distinguishing predictive capability remains comparatively limited.

Only a relatively small number of studies have employed explicitly path-distinguishing loading schedules, including strain-rate jumps [7,30,79], multi-directional deformation [6], multi-stage loading [80], cyclic or reversal loading [81], and non-constant thermo-mechanical histories [73].

A representative example is provided by the work of Pei et al. [79], who compared several constitutive formulations under strain-rate-jump conditions in a superplastic magnesium alloy. Although the artificial neural network achieved the highest fitting accuracy under monotonic loading, the ISV-based formulation produced superior predictions during strain-rate transitions because deformation history was explicitly retained through evolving internal states. The study illustrates a broader principle that is directly relevant to SPF constitutive modeling: predictive performance under monotonic loading does not necessarily indicate predictive capability under path-dependent conditions.

Table 3 summarises representative validation strategies reported in the reviewed literature. A clear trend emerges: while constitutive memory capacity generally increases from Type II to Type IV architectures, the number of studies employing correspondingly demanding path-distinguishing validation procedures remains comparatively limited. As a result, the advantages of higher-memory constitutive architectures are often inferred from their structure rather than demonstrated through systematic validation.

The evidence reviewed here therefore suggests that constitutive memory capacity and demonstrated path-dependent predictive capability should not be treated as equivalent concepts. Distinguishing between architectural potential and experimentally validated performance provides a more reliable basis for comparing constitutive models intended for SPF applications.

### 4.3. Future Directions

#### 4.3.1. Hybrid and Physics-Informed Memory Architectures

The preceding discussion suggests that future constitutive development should not aim to maximize memory capacity indiscriminately, but rather to retain the deformation-history information necessary for the intended SPF prediction task. From this perspective, hybrid constitutive architectures that combine physically interpretable internal state variables with data-driven learning components appear particularly promising.

Recent studies have demonstrated the feasibility of integrating machine learning with internal-state-variable frameworks. He and Chen developed a thermodynamically consistent approach in which internal state variables are inferred from recurrent neural-network representations [82]; although originally demonstrated on soil materials, the thermodynamic-ML-ISV architecture is material-agnostic and directly applicable to titanium-alloy constitutive modeling. Meyer and Ekre further showed that neural networks can be embedded within constitutive evolution laws while preserving thermodynamic consistency [83]. Such approaches offer a potential pathway for combining the interpretability and FEM compatibility of Type III architectures with the enhanced sequence-learning capability of Type IV models.

Physics-informed machine learning provides a complementary direction. By incorporating physical constraints directly into model architecture or training procedures, these approaches improve robustness while reducing dependence on large path-resolved datasets [84,85]. For SPF applications, where experimental data are often costly to obtain, physics-informed frameworks may provide an effective means of representing complex deformation histories without sacrificing physical consistency or practical implementability.

#### 4.3.2. Path-Distinguishing Validation and Memory-Aware Evaluation

As Section 4.2.2 highlighted, constitutive memory is frequently assumed rather than explicitly validated. Future progress therefore depends not only on developing new constitutive architectures but also on establishing validation strategies capable of assessing deformation-history representation directly.

Particular value may be found in benchmark datasets incorporating strain-rate jumps, multi-stage deformation, non-proportional loading, and other path-distinguishing conditions [6,7,30,73,80]. Such datasets would enable constitutive architectures with different memory capacities to be evaluated on a common basis and would provide a more rigorous assessment of path-dependent predictive capability than conventional monotonic testing alone.

More broadly, constitutive-model assessment may benefit from moving beyond fitting accuracy as the sole performance criterion. Models intended for SPF applications should increasingly be evaluated according to their ability to distinguish alternative deformation histories, reproduce evolving material states, and retain the information required for the prediction task of interest. From a memory-architecture perspective, the ultimate objective is not to maximize constitutive memory but to determine the amount of memory that is necessary, measurable, and practically useful for reliable SPF process prediction.

## 5. Conclusions

This review examined constitutive modeling for titanium-alloy superplastic forming (SPF) from the perspective of deformation-history representation. A memory-architecture framework was proposed to classify constitutive models according to how prior thermo-mechanical information is retained and utilized during deformation. Four broad constitutive architectures were identified: stateless models, implicit or projected memory models, reduced-order memory models, and high-dimensional memory models.

The analysis suggests that differences among constitutive models extend beyond their mathematical formulation or predictive accuracy. More fundamentally, they differ in the amount and type of deformation-history information available to influence the current material response. From this perspective, constitutive modeling may be viewed as a problem of history representation. The review further indicates that the practical value of constitutive memory depends strongly on the prediction objective. Conventional Type I–II architectures are often sufficient for monotonic flow-stress prediction, whereas Type III architectures become increasingly important for thickness evolution, microstructural evolution, and damage-related SPF simulations. Type IV architectures offer greater sequence sensitivity but currently face challenges associated with data requirements, interpretability, and industrial implementation.

The memory-architecture perspective provides a common framework for comparing empirical, physically based, and data-driven constitutive approaches. More importantly, it suggests that constitutive-model selection should be guided not only by fitting accuracy but also by the amount of deformation-history information required for the intended prediction task. The central challenge for future SPF constitutive modeling is therefore not to maximize constitutive memory, but to retain the history information that is necessary, measurable, and practically useful for reliable process prediction.

## Figures and Tables

**Table 1:** Memory-architecture-based classification of constitutive models.

Type	Memory Architecture	Memory Representation	Generic Constitutive Form	Representative Examples
I	Stateless	None	$\sigma =f(\dot{\varepsilon },T)$	Basic power-law, steady Arrhenius [32,33]
II	Implicit/Projected Memory	Strain-dependent descriptors	$\sigma =f(\dot{\varepsilon },T,P\left(\varepsilon \right))$	Strain-compensated Arrhenius, modified JC [27,31,34]
III	Reduced-Order Memory	Internal state variables	$\sigma =f(\dot{\varepsilon },T,S)$	ISV, DRX, grain-size, damage models [7,13,35,36]
IV	High-Dimensional/ Explicit Memory	Latent states, physical fields, sequences	$\sigma =f(\dot{\varepsilon },T,M)$	LSTM, Transformer, microstructure-resolved models [30,37,38]

**Table 2:** Cost–benefit comparison of constitutive memory architectures.

Type	Memory Capacity	Principal Benefit	Main Trade-Off
I	None	Simple and computationally efficient	No path representation
II	Low	Improved accuracy under monotonic loading	Limited path distinguishability
III	Moderate	Physically interpretable path representation	Additional calibration and state evolution
IV	High	Strong sequence and history sensitivity	High data and computational requirements

**Table 3:** Representative validation strategies reported for constitutive models with different memory architectures.

Study	Material	Type	Validation	Path-Sensitive?
Arrhenius [27,31,42]	Ti	II	Monotonic	No
Johnson–Cook [53]	Ti	II	Monotonic	No
ISV models [13,17,18]	Ti	III	Monotonic	No *
Wang [7]	Ti	III	Rate jump	Yes
Guo [6]	Ti	III	Multi-path	Yes
Zhang [80]	Ti	III	Multi-stage	Yes
Bambach [30]	Al	IV	Rate jump	Yes
Gao [73]	Al	IV	Variable history	Yes
Li [81]	Metal	IV	Reversal loading	Yes
Wang [75]	Metal	IV	Monotonic	No *
Pei [79]	Mg	III	Rate jump	Yes

* Architecture capable but not experimentally demonstrated.

## Data Availability

No new data were created or analyzed in this study. Data sharing is not applicable to this article.

## References

[1] Li Y., Jiang S., Peng P., Zhang J., Yang S., Lu Z., Jia Y. (2025). Superplastic deformation and macrozone behavior of the Ti60 high-temperature titanium alloy. J. Alloys Compd..

[2] Yadav P., Saxena K.K., Sehgal S., Singh T., Bahl S. (2022). Hot deformation behaviour of Ti alloys: A review on physical simulation and deformation mechanisms. Proc. Inst. Mech. Eng. Part E J. Process Mech. Eng..

[3] Liu Y., Yang Q., Tian W., He T., Cui L., Liu X., Liu S., Wang K. (2025). Enhancing Workability Through Adjusting Element Content: A Study on the Hot Deformation Behavior of a Modified Titanium Alloy. Adv. Eng. Mater..

[4] Han Y., Dong E.T., Yu W., Shi J.X., Cheng L. (2025). Deformation Mechanism in Ti–6Al–4V Seamless Tube with Microstructure Formed upon Continuous Cooling to Two-Phase Region. Phys. Met. Metallogr..

[5] Wang Y., Li Z., Wang H., Hou M., Yu K., Xu Y., Xiao H. (2024). Flow behavior and dynamic recrystallization mechanism of a new near-alpha titanium alloy Ti-0.3Mo-0.8Ni-2Al-1.5Zr. J. Mater. Res. Technol..

[6] Guo S., Yan S., Huang L., Liu K., Li C. (2025). A strain-path dependent unified constitutive model of titanium alloy coupling coarse grain subdivision and recrystallization: Application to multi-directional hot deformation. Int. J. Plast..

[7] Wang K., Song K., Zhao J., Cui S., Peng C., Wang X., Wang L., Liu G. (2022). Physically-based constitutive models for hot gas pressure forming of laser-welded titanium alloy blank. J. Manuf. Process..

[8] Sun Q., Zhou J., Peng J., Chen X. (2022). Superplasticity study of TA15 alloy based on variable m value method. Rare Met. Mater. Eng..

[9] Sun Q.J., Wang G.C., Li M.Q. (2012). Enhanced the superplasticity in Ti-6.5Al-2Zr-1Mo-1V alloy by a two-step deformation method. Mater. Des..

[10] Wang G., Fu M.W. (2007). Maximum m superplasticity deformation for Ti–6Al–4V titanium alloy. J. Mater. Process. Technol..

[11] Lu T., Dan Z., Li T., Dai G., Sun Y., Guo Y., Li K., Yi D., Chang H., Zhou L. (2022). Flow softening and microstructural evolution of near *β* titanium alloy Ti-35421 during hot compression deformation in the *α*+*β* region. J. Mater. Res. Technol..

[12] Sun T., Sun L., Teng H., Liu W., Wang R., Zhao X., Zhou J. (2024). Constitutive Model and Microstructure Evolution of Ti65 Titanium Alloy. Materials.

[13] Feng P., Wang B., Yang C., Han Y., Jin K. (2023). Internal–State–Variable Based Unified Viscoplastic Constitutive Modeling of TC11 Titanium Alloy and Its Microstructure Evolution Simulation. Metall. Mater. Trans. A.

[14] Yang J., Wu J., Yang D., Wang Q., Wang K., Zhang Z., Wang M., Muzamil M. (2020). A Modified Constitutive Model with Grain Rotation for Superplastic Forming of Ti–6Al–4V Alloy. J. Eng. Mater. Technol..

[15] Dear J., Zhang R., Shi Z., Lin J. (2025). A novel data-driven machine learning approach for improved strain rate control in thermomechanical testing of sheet metals. Eng. Appl. Artif. Intell..

[16] Hussain A., Sakhaei A.H., Shafiee M. (2024). Machine learning-based constitutive modelling for material non-linearity: A review. Mech. Adv. Mater. Struct..

[17] Alabort E., Putman D., Reed R. (2015). Superplasticity in Ti–6Al–4V: Characterisation, modelling and applications. Acta Mater..

[18] Liu Y., Sun C., Li Z., Zhao B., Zhu X., Xu S., Qian L. (2025). Superplastic constitutive modeling of TA32 alloy with two-phase characteristics. Int. J. Mech. Sci..

[19] Akula S.P., Ojha M., Rao K.L., Gupta A.K. (2023). A review on superplastic forming of Ti-6Al-4V and other titanium alloys. Mater. Today Commun..

[20] Frost H.J., Ashby M.F. (1982). Deformation-Mechanism Maps: The Plasticity and Creep of Metals and Ceramics.

[21] Nieh T., Wadsworth J., Sherby O.D. (1997). Superplasticity in Metals and Ceramics.

[22] Yang J., Wu J., Zhang Q., Han R., Wang K. (2020). The simple hyperbolic-sine equation for superplastic deformation and parameters optimization. J. Mater. Res. Technol..

[23] Ma L., Wan M., Li W., Shao J., Han X., Zhang J. (2022). On the superplastic deformation mechanisms of near-*α* TNW700 titanium alloy. J. Mater. Sci. Technol..

[24] Li L., Liu W., Wang K., Wang D., An Q., Liu G. (2025). Recrystallization and deformation mechanisms of network-structured TiBw/(TA15-Si) composite. Chin. J. Aeronaut..

[25] Yang J., Wu J., Xie H., Li Z., Wang K. (2023). Mechanism of continuous dynamic recrystallization of Ti–6Al–4V alloy during superplastic forming with sub-grain rotation. Trans. Nonferrous Met. Soc. China.

[26] Li L., Li M. (2018). Grain size model for continuous dynamic recrystallization of titanium alloy in hot deformation. Sci. China Technol. Sci..

[27] Mosleh A.O., Mikhaylovskaya A.V., Kotov A.D., Kwame J.S., Aksenov S.A. (2019). Superplasticity of Ti-6Al-4V Titanium Alloy: Microstructure Evolution and Constitutive Modelling. Materials.

[28] Wang L., Li W., Luan S., Jin P., Wang J., Ren Q., Zhu L. (2023). Study on hot deformation behavior of as-cast Ti-5Al-5Mo-5V-1Cr-1Fe titanium alloy in (*α*+*β*) phase region. Mater. Today Commun..

[29] Feng X., Hu L., Sun Y. (2021). Optimization of the hot working parameters of a nickel-based superalloy using a constitutive-dynamic recrystallization model and three-dimensional processing map. J. Mater. Sci..

[30] Bambach M., Gerster S., Herty M. (2021). Online data assimilation of a hybrid flow stress model by particle filtering. CIRP Ann..

[31] Gao F., Li W., Meng B., Wan M., Zhang X., Han X. (2017). Rheological law and constitutive model for superplastic deformation of Ti-6Al-4V. J. Alloys Compd..

[32] Backofen W.A. (1964). Metallurgical Aspects of Ductile Fracture. Fracture of Engineering Materials.

[33] Langdon T.G. (1991). The physics of superplastic deformation. Mater. Sci. Eng. A.

[34] Sun W., Yang X., Liu S., Huang X., Wang B., Ji H. (2025). Thermal deformation behavior and constitutive model of TC4 titanium alloys at hot tensile process. Mater. Today Commun..

[35] Cai Z., Li Y., Xu Y., Chen J., Li X., Ma G., Li D. (2025). Unified constitutive modeling for hot tensile behavior of TC4 alloy with and without diffusion bonding joint. J. Mater. Res. Technol..

[36] Li L., Liu J., Ding N., Li M. (2023). Substructure evolution in two phases based constitutive model for hot deformation of TC18 in *α*+*β* phase region. Chin. J. Aeronaut..

[37] Hannequart P., Peigney M., Caron J. (2019). A micromechanics-based model for polycrystalline Ni–Ti wires. Smart Mater. Struct..

[38] Zhang S., Shu C., Xiang G., Zhang J., Tao X., Zhu X., Gu Q., Hua L., Xue S., Yao Z. (2026). Physically interpretable flow behavior prediction of 42CrMo steel via CA-simulation-informed multimodal deep learning. Mater. Sci. Eng. A.

[39] Chen P., Li J., Li C., Yu Y., Cai J. (2025). Research on the hot-temperature rheological behavior and microstructural evolution of TA18 titanium alloy. Mater. Today Commun..

[40] Mosleh A., Mikhaylovskaya A., Kotov A., Pourcelot T., Aksenov S., Kwame J., Portnoy V. (2017). Modelling of the Superplastic Deformation of the Near-*α* Titanium Alloy (Ti-2.5Al-1.8Mn) Using Arrhenius-Type Constitutive Model and Artificial Neural Network. Metals.

[41] Santosh S., Sampath V., Mouliswar R.R. (2025). Elevated Temperature Deformation Behavior of Novel NiTiAg Shape Memory Alloys: A Comparison Between Various Constitutive Models and Experimental Flow Curves. J. Mater. Eng. Perform..

[42] Wang X., Liu P., Liang C., Lu T., Feng T., Niu H., Dong Y., Liu X. (2024). Investigation on the thermal deformation mechanisms and constitutive model of Ti-55511 titanium alloy. J. Mater. Res. Technol..

[43] Wu T., Chen M., Dong H., Wen X., Sui Q. (2025). Research on the deformation behavior at elevated temperature of Ti-6Al-4V titanium based on the recurrent neural network. J. Mater. Eng. Perform..

[44] Zhang H., Zhang Y., Huang Y., Wang B., Wei W., Qin S., Zhou H., Liu J. (2024). The thermal deformation behavior and processing map of TC9 titanium alloy. J. Mater. Res. Technol..

[45] Xu Q., Li W., Yin Y., Zhou J. (2021). Closure Behavior of the Artificial Gas Pore inside the As-Cast Ti6Al4V Alloy during HIP: Constitutive Modeling and Numerical Simulation. Metals.

[46] Yang X., Wang Y., Dong X., Peng C., Ji B., Xu Y., Li W. (2021). Hot deformation behavior and microstructure evolution of the laser solid formed TC4 titanium alloy. Chin. J. Aeronaut..

[47] Pak H., Sim K.H., Ri Y.C., Kim R.S., Ri J.H. (2023). Comparisons of phenomenological and physically based constitutive models for Ti–6Al–2Zr–2Sn–3Mo–1.5Cr–2Nb alloy. Appl. Phys. A.

[48] Lee J., Lee Y., Lee D. (2025). High-temperature deformation mechanism of Ti–5Mo–4Fe metastable beta titanium alloy: Synergistic effect of strain rate sensitivity, Z-parameter, and activation energy. J. Mater. Res. Technol..

[49] Zhao H., Wu J., He H., Li X., Wang L., Lou H., Liu K., Ruan X. (2024). A comparative study of hot tensile deformation behavior of 6016 aluminum alloy under LSTM neural network and arrhenius model. Mater. Res. Express.

[50] Han X., Yang J., Li J., Wu J. (2022). Constitutive Modeling on the Ti-6Al-4V Alloy during Air Cooling and Application. Metals.

[51] Imran S.M., Li C., Lang L., Guo Y., Mirza H.A., Haq F., Alexandrova S., Jiang J., Han H. (2022). An investigation into Arrhenius type constitutive models to predict complex hot deformation behavior of TC4 alloy having bimodal microstructure. Mater. Today Commun..

[52] Yu R., Li X., Li W., Chen J., Guo X., Li J. (2021). Application of four different models for predicting the high-temperature flow behavior of TG6 titanium alloy. Mater. Today Commun..

[53] Zhu J., Zhi H., Huang T., Ding N., Yan Z. (2025). Research on the Johnson–Cook constitutive model and failure behavior of TC4 alloy. Metals.

[54] Liu H., Wang Y., Li J., Cheng Y., Sun D., Jiang S., Han L., Wang Y. (2025). A study of Johnson–Cook model coefficient corrections in the cryogenic machining of Ti-5Al-2.5Sn alloy. Eng. Fract. Mech..

[55] Seddik R., Rondepierre A., Prabhakaran S., Morin L., Favier V., Palin-Luc T., Berthe L. (2022). Identification of constitutive equations at very high strain rates using shock wave produced by laser. Eur. J. Mech. A Solids.

[56] Zhou T., He L., Feng Z., Tian P., Du F., Zou Z., Zhou X. (2022). Inverse identification of material constitutive parameters based on co-simulation. J. Mater. Res. Technol..

[57] Dou W., Xu Z., Hu H., Huang F. (2021). A generalized plasticity model incorporating stress state, strain rate and temperature effects. Int. J. Impact Eng..

[58] Li P., Yu R., Yan S., Zhang C., Wang Y., Yang L., Xue K. (2024). Study on deformation behavior of Ti60 alloy based on multi-physics coupling. Mater. Today Commun..

[59] Palanisamy N.K., Lorphevre E.R., Gobert M., Briffoteaux G., Tuyttens D., Arrazola P., Ducobu F. (2022). Identification of the parameter values of the constitutive and friction models in machining using EGO algorithm: Application to Ti6Al4V. Metals.

[60] Wang K., Wang L., Zheng K., He Z., Politis D.J., Liu G., Yuan S. (2020). High-efficiency forming processes for complex thin-walled titanium alloys components: State-of-the-art and perspectives. Int. J. Extrem. Manuf..

[61] Buzolin R.H., Lasnik M., Krumphals A., Poletti M.C. (2021). A dislocation-based model for the microstructure evolution and the flow stress of a Ti5553 alloy. Int. J. Plast..

[62] Wu Y., Wu D., Ma J., Xiao W., Zheng K., Chen M. (2021). A physically based constitutive model of Ti-6Al-4 V and application in the SPF/DB process for a pyramid lattice sandwich panel. Arch. Civ. Mech. Eng..

[63] Niu H.T., Gao P.F., Li H.W., Zhan M. (2025). Mechanism and modelling of the electroplastic effect in titanium alloy: From the perspective of dislocation slip. Int. J. Plast..

[64] Wang S., Deng X., Gao P., Ren Z., Wang X., Feng H., Zeng L., Zhang Z. (2024). Physical constitutive modelling of hot deformation of titanium matrix composites. Int. J. Mech. Sci..

[65] Ma L., He D., Yang B., Han X., Zhang J., Li W. (2025). Construction and application of a physically-based constitutive model for superplastic deformation of near-*α* TNW700 titanium alloy. J. Mater. Res. Technol..

[66] Wang S., Yang J., Hu P., Wang X., Wang Z., Wang Q., Feng R., Jin B., Wang K. (2025). Research on the high-temperature deformation constitutive model of La-TZM alloy. J. Mater. Sci..

[67] Jiang X., Du J., Fan Y., Huang J., Pan F. (2019). One-Dimensional Macroscopic Constitutive Model for Ratcheting of Superelastic Shape Memory Alloys. J. Eng. Mech..

[68] Nazzal M.A., Khraisheh M.K., Abu-Farha F.K. (2007). The effect of strain rate sensitivity evolution on deformation stability during superplastic forming. J. Mater. Process. Technol..

[69] Chang C., Yang J. (2024). The Constitutive Equation-Based Recrystallization Mechanism of Ti-6Al-4V Alloy during Superplastic Forming. Coatings.

[70] Pak H.S., Sim K.H., Ri B.H., Jang G.S. (2025). Microstructure-based constitutive modeling of flow stress behavior of Ti-6Al-2Zr-2Sn-3Mo-1.5Cr-2Nb alloy at thermo-mechanical processing conditions. Appl. Phys. A.

[71] Buzolin R.H., Lasnik M., Krumphals A., Poletti M.C. (2021). Hot deformation and dynamic *α*-globularization of a Ti-17 alloy: Consistent physical model. Mater. Des..

[72] Yu C., Jiang H.M., Song D., Zhu Y., Kang G. (2023). A multi-scale diffusional-mechanically coupled model for super-elastic NiTi shape memory alloy wires in hydrogen-rich environment. Int. J. Plast..

[73] Gao C., Wen H., Long J., Jin J., Tang X., Wang X., Deng L., Gong P., Zhang M. (2025). Enhancing constitutive modeling and workability analysis via deformation history-informed recurrent neural networks: A case study on 2024 aluminum alloy. J. Mater. Process. Technol..

[74] Li J., Guan Z., Chen J., Jin H. (2025). A long short-term memory-based constitutive modeling framework for capturing strain path dependence in plastic deformation. Mech. Mater..

[75] Wang X., Huo Y., Wang Z., Yan Z., Yu W., Ma K., Chen H., Sun Y., Crabbe M.J.C., Yue X. (2025). Comparison of modified Arrhenius, modified Zerilli–Armstrong, and long short-term memory models for predicting hot deformation behavior of NiAlCrFeMo high-entropy alloy. J. Mater. Eng. Perform..

[76] Wen H., Wang S., Jin J., Wang X., Tang X., Zhang Y., Deng L., Gong P., Li D., Ning B. (2024). Deep learning-based modeling of the strain rate-dependent thermomechanical processing response for a novel HIPed P/M nickel-based superalloy. J. Mater. Process. Technol..

[77] Wu T., Chen M. (2025). An approach for hot deformation behavior prediction based on the adaptive sparse self-attention mechanism. Mater. Today Commun..

[78] Alabort E., Kontis P., Barba D., Dragnevski K., Reed R.C. (2016). On the mechanisms of superplasticity in Ti–6Al–4V. Acta Mater..

[79] Pei Y., Li L., Yu M., Wei E., Zhao M., Teng B. (2025). Comparison and evaluation of different constitutive models for predicting the hot deformation behavior of Mg-Gd-Y-Zr alloy. J. Magnes. Alloys.

[80] Zhang S., Lin Y.C., Ling Y., He D., Chen M., Wan M., Wu G., Zeng N., Zhang H., Naseri M. (2025). Modeling Flow Behaviors and Microstructure Evolution of Ti55511 Alloy During the Double-Stage Hot Deformation Process Utilizing Machine Learning Algorithm. Met. Mater. Int..

[81] Li Q., Cinbiz M.N., Zhang Y., He Q., Beausoleil G., Li J. (2023). Robust deep learning framework for constitutive relations modeling. Acta Mater..

[82] He X., Chen J. (2022). Thermodynamically consistent machine-learned internal state variable approach for data-driven modeling of path-dependent materials. Comput. Methods Appl. Mech. Eng..

[83] Meyer K.A., Ekre F. (2023). Thermodynamically consistent neural network plasticity modeling and discovery of evolution laws. J. Mech. Phys. Solids.

[84] Masi F., Stefanou I., Vannucci P., Maffi-Berthier V. (2021). Thermodynamics-based artificial neural networks for constitutive modeling. J. Mech. Phys. Solids.

[85] Eghtesad A., Tan J., Fuhg J.N., Bouklas N. (2024). NN-EVP: A physics informed neural network-based elasto-viscoplastic framework for predictions of grain size-aware flow response. Int. J. Plast..

